# Real-Time Analysis of Predictors of COVID-19 Infection Spread in Countries in the European Union Through a New Tool

**DOI:** 10.3389/ijph.2022.1604974

**Published:** 2022-10-06

**Authors:** Aniko Balogh, Anna Harman, Frauke Kreuter

**Affiliations:** ^1^ School of Social Sciences and Mannheim Business School, University of Mannheim, Mannheim, Germany; ^2^ TÁRKI Social Research Institute, Budapest, Hungary; ^3^ Joint Program in Survey Methodology, University of Maryland, College Park, MD, United States; ^4^ Statistics and Data Science in Social Sciences and the Humanities at the Ludwig-Maximilians-University of Munich, Munich, Germany

**Keywords:** machine learning, time series cross-validation, interactive visualization, COVID-19 prediction, comparative analyses, COVID-19 non-pharmaceutical interventions, social epidemiology, COVID-19 virus variants

## Abstract

**Objectives:** Real-time data analysis during a pandemic is crucial. This paper aims to introduce a novel interactive tool called Covid-Predictor-Tracker using several sources of COVID-19 data, which allows examining developments over time and across countries. Exemplified here by investigating relative effects of vaccination to non-pharmaceutical interventions on COVID-19 spread.

**Methods:** We combine >100 indicators from the Global COVID-19 Trends and Impact Survey, Johns Hopkins University, Our World in Data, European Centre for Disease Prevention and Control, National Centers for Environmental Information, and Eurostat using random forests, hierarchical clustering, and rank correlation to predict COVID-19 cases.

**Results:** Between 2/2020 and 1/2022, we found among the non-pharmaceutical interventions “mask usage” to have strong effects after the percentage of people vaccinated at least once, followed by country-specific measures such as lock-downs. Countries with similar characteristics share ranks of infection predictors. Gender and age distribution, healthcare expenditures and cultural participation interact with restriction measures.

**Conclusion:** Including time-aware machine learning models in COVID-19 infection dashboards allows to disentangle and rank predictors of COVID-19 cases per country to support policy evaluation. Our open-source tool can be updated daily with continuous data streams, and expanded as the pandemic evolves.

## Introduction

A novel coronavirus originated from China [1] that causes the COVID-19 disease has escalated rapidly around the Globe [2], resulting in fundamental life-changing effects. As of 30 July 2022, the virus has infected more than 590 million individuals, caused about 64 million deaths [3]. Every variant changes the course and contours of the COVID-19 pandemic. Nations navigate this dynamic political, economic, and social environment, responding to the steam of challenges with a range of approaches that reflect the complex diversity of polities and circumstances.

After more than 2 years of employing a diverse set of non-pharmaceutical interventions (NPI) governments are eager to evaluate the effectiveness of their measures and compare their strategies to other countries. Seeking to assist decision-making around the identification of COVID-19 infection predictors, we built a data-driven [4, 5] interactive visualization and analysis tool called Covid-Predictor-Tracker using a wide range of COVID-19 related time series data. The Covid-Predictor-Tracker—available at https://corona.stat.uni-muenchen.de/covid_FI/ - allows the retrospective time series analyses by country and across countries of COVID-19 infections, as a function of individual behaviors, country-specific characteristics, and NPI measures over time.

Several tools exist for various aspects of the COVID-19 pandemic. Most provide exploratory features like Our World in Data [6] and the Johns Hopkins Cornonavirus Resource Center [3, 7] with a global perspective, or local ones like the Dutch COVID-19 Dashboard [8] and COnVIDa [9]. Few provide model-based analytical elements like the COVID-19 Spread Mapper [10] with log-linear modeling and epiMOX [11] with a compartment model and what-if analysis simulating different epidemic trends.

While nearly all dashboards report epidemiological indicators according to a descriptive assessment of 158 public online COVID-19 dashboards [12], indicators on social, economic factors and behavioral insights are rarely reported (4.4%, 1.3%, respectively). Our tool stands out with an extensive coverage of a wide range of factors and with its model-based analytical approach, incorporating diverse fields relevant to the virus spread.

**FIGURE 1:**
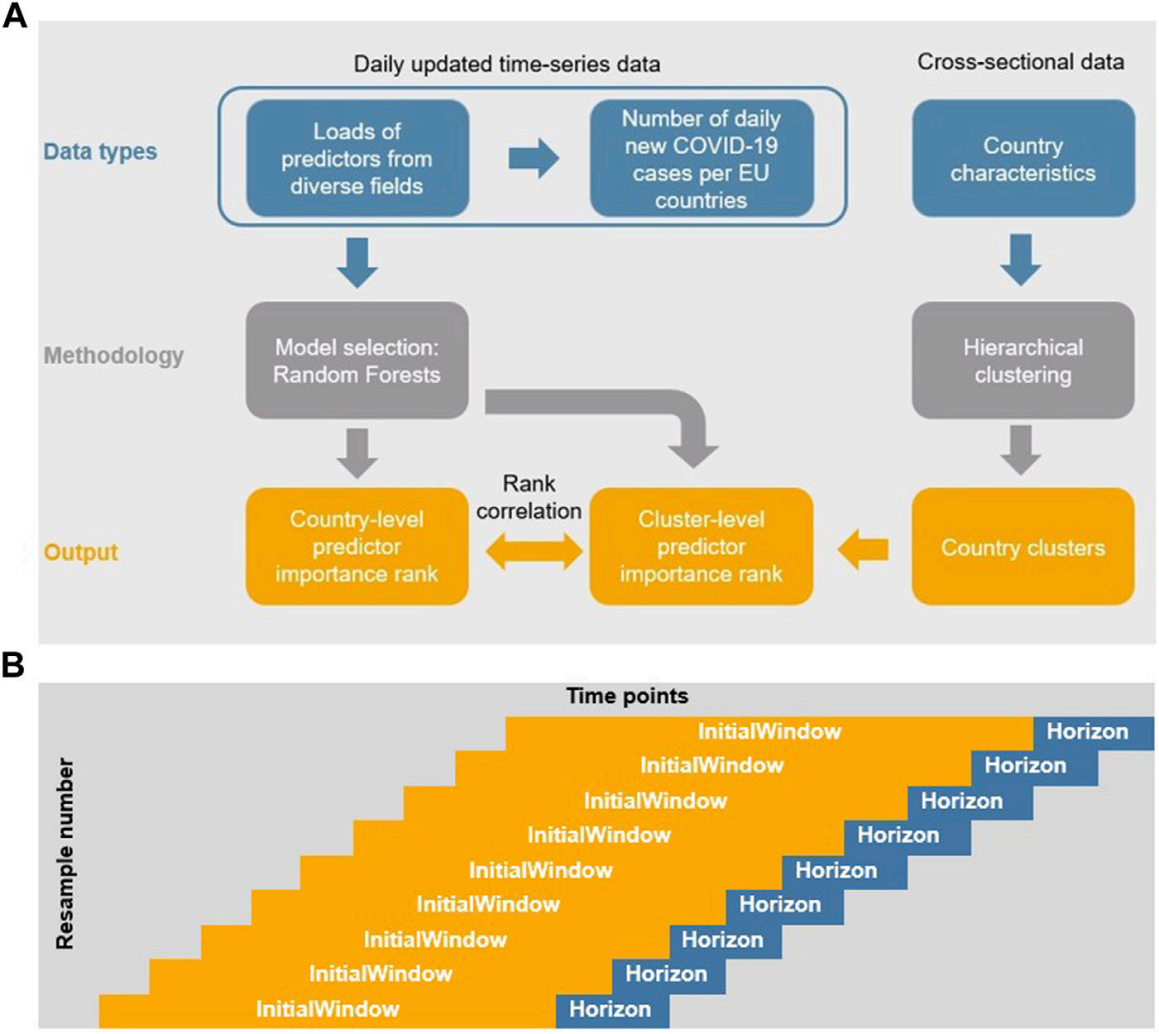
Flow of data and models and Schema of the rolling forecasting origin method. **(A)** Flow of data and models (Selected countries of the European Union, 2020–2022). **(B)** Schema of the rolling forecasting origin method (Covid-Predictor-Tracker, Selected countries of the European Union, 2020–2022).

In our modeling approach (see Figure 1A) we started with a machine learning random forest (RF) algorithm (see also [13–15]) applied to a time series database of COVID-19-related variables to find the most important variables predicting COVID-19 spread across the EU countries. These ensemble of learning methods for classification or regression [16] combine several regression trees in a way that each tree depends on the values of a random vector sampled independently and with the same distribution for all the trees. The importance of a single variable can be assessed [17]. At this step we considered a wide range of variables related to COVID-19 spread, including behavioral responses like self-reported mask usage and the frequency of direct contact [18], a meteorological factor [19], vaccination [20] rates, share of COVID-19 variants [21] and NPIs [22], as literature suggests. While the use of machine learning models in COVID-19 prediction is not unique, Alali [23] points out, most of the machine learning models do not consider the time dependency of data series. In contrast, our RF approach underlines the importance of the time dependency in COVID-19 data, capturing it by applying time-series cross-validation during the RF training phase.

As Ying [24] and Farmer [25] highlight, it is crucial to reveal the relationship between the disease spread and socioeconomic and health indicators across regions. To do so, we included age and gender distribution, health expenditure, and cultural participation in our model. As a second step, agglomerative hierarchical clustering [26] was used to form relevant groups of countries based on these time-constant country characteristics. Agglomerative hierarchical clustering starts with every country representing a single cluster and, in every step of the algorithm, one pair of clusters, the one with the smallest intergroup dissimilarity is merged into one group. The algorithm stops when there is only one cluster left, this one contains all the countries.

As a third step, we calculated Spearman’s rank correlations [27] between the predictor importance ranks of each country and the predictor importance rank of the relevant country–cluster assessing monotonic relationships between these variables. To get the latter measures, a RF model was run on each country cluster (omitting country borders within clusters) in addition to the same RF models for each single country. This way we can examine for each country whether its most important predictors of COVID-19 daily new infections are typical for countries with similar country characteristics or not.

We elaborate on the customization of the RF method and on hierarchical clustering in the following section after we describe the data sources used. We then share some selected findings focusing on country comparisons. We display and discuss the most important predictors of the spread of the COVID-19 infection for the time frame between February 2020 and January 2022. We close with some suggestions for improvements of the Covid-Predictor-Tracker tool, however in its existing form it can already help public health authorities to examine the effect of interventions/campaigns related to other influential factors, while controlling for basic country characteristics.

## Methods

We used nine data sources to build two integrated databases. A time-constant, cross-sectional database on general country characteristics, and a time-varying time series database of COVID-19-related variables. We describe the (automated) data collection and updating processes, as well as the database preparation, to include daily updates as the pandemic continues. The publicly available data streams are captured *via* APIs (Application Programming Interfaces) or csv (comma-separated value) files from provider homepages. We selected data sources based on validity [28–32], accessibility, regularity of updates, and availability since the start of the pandemic [33].

### Data Sources and Collection

The time-constant country characteristics are extracted using an API provided by Eurostat. The most recent available data were used to capture country population characteristics such as age and gender, as well as total population size [34], health expenditures [35], and cultural participation [36]. Pre-COVID-19 cultural participation indicators included the percentage of 16-year-old and older, under 30, and over 75 year-old who did not attend any broadly defined cultural event in the last 12 months.

The time-varying covariates and outcome information come from six different online sources. Daily average temperatures are obtained from the National Centers for Environmental Information [37] using the rnoaa R package [38].

Country-specific response measures are downloaded from the homepage of the European Centre for Disease Prevention and Control (ECDC) [39] using the data.table R package [40]. Because links to this database change from time to time, we extracted the html code of the homepage with the rvest R package [41]. The share of COVID-19 variants among the newly registered cases are downloaded as a csv file from this homepage [39].

Number of new infections, deaths, and recoveries related to COVID-19 are extracted from the COVID-19 Data Repository [3] by the Center for Systems Science and Engineering at Johns Hopkins University (JHU CCSE) using the coronavirus R package [42].

Behavioral responses to the pandemic were captured in the Global COVID-19 Trends and Impact Survey (CTIS) [43, 44]. Astley et al. (2021) [45] evaluated internal and external validity of the CTIS data. We used the open API [44] to download country-specific aggregates of “mask usage,” “direct contact,” and “reported COVID-like illness symptoms.”

The data about “new and total number of vaccinations” and “proportion of vaccinated people” [6] stem from Our World in Data access with the data.table R package [40].

To enable effective automatic updating and quality control, a back-check is programmed. Anytime the database is updated, a list is created automatically for the overlapping periods showing the differences between the newly downloaded data and the previous version. This feature allows users to follow the corrections made by the data providers. All code can be found on GitHub at https://github.com/covidrealtime/covidrealtime, and is described in the Supplementary File. All variables were checked for implausible and missing values. Standardizations and variable transformations were used to combine all time-constant country-specific variables together in one database, and all time-changing variables in another. The full list of variables can be found in the Supplementary File.

### Model Selection

Following Shmueli [46] and his conception framework we use a random forest (RF) machine learning approach, capturing the association between outcome and predictors. Because our focus is to reveal the effects of many predictors, rather than to forecast a single time series, ARIMA, ARCH/GARCH models are less ideal. Shang et al. [47] argues, Vector Autoregression, a forecasting algorithm for multivariate time-series often used when two or more time-series influence each other, is less suited for epidemiological outcomes. Kane et al. [13] show that RF outperformed ARIMA time series models for prediction of avian influenza H5N1 outbreaks. Yeşilkanat [14] achieved good results for COVID-19 when used for spatial-temporal prediction on worldwide daily cases of COVID-19 applying RF. Cobb et al. [15] saw RF outperform other statistical analyses when examining the effect of social distancing on the compound growth rate of COVID-19. As Shmueli [46:292] states, “Newly available large and rich datasets often contain complex relationships and patterns that are hard to hypothesize,” and assumptions on variable distribution would be problematic as well.

### Our Model

We use RF to predict the permutation feature importance of many predictors of the change in daily confirmed new COVID-19 cases over 14 days across the countries in the EU. Smoothing was implemented with 7-day rolling averages. The change in the number of cases is proportional to population size. Repeated permutation (variable importance) results can be unstable, so we averaged the importance measures over repetitions of 5 to stabilize the rank of feature importance.

We used multilevel models with time points nested within each country, following the approach of Chakraborti et al. [48], who compared the five continents exploring determinant factors of the present pandemic comparing the results of five runs of their RF model. Data were split by countries generating a list with countries at the first level and RF was implemented throughout the list *via* functional programming.

To evaluate the effect of country characteristics on feature importance, we run the same RF models on the clusters formed by covariate combinations, omitting country borders. As countries within clusters are similar to each other, we can neglect country-level case dependency in case of country clustering. All predictors were standardized before we added them into the model. None of the bivariate correlations of the predictors were above 0.7, thus conditional forests were not needed [49]. Average temperature, COVID-like illness, mask coverage, and direct contact variables were smoothed with a 7-day rolling average.

### Time-Series Cross-Validation

To honor the time-dependent structure of the data when forming out training and test data we used the rolling forecasting origin technique, introduced by Hyndman/Athanasopolous [50], *via* the R package caret [51]. In this procedure, there are a series of test sets, each consisting of fixed lengths of observations (see Figure 1B) [52]. An advantage of this approach is that “corresponding training set consists only of observations that occurred prior to the observation that forms the test set. Thus, no future observations can be used in constructing the forecast” [50:84].

The number of consecutive values in each training set sample (called initialWindow in R package caret and in Figure 1B) is set to 28 days in order to cover a period long enough to contain enough time to possibly show an effect of a response measure considering the combination from the incubation period of COVID-19 with a median 4.5–5.8 days (95% CI) [53], and the test delay (time until doctor visit and test evaluation time) [54].

The number of consecutive values in the test set sample (called Horizon in caret and Figure 1B) is 5 to allow for a relatively high number of resamples without “running out” of the time series over time. Our model used between 246 and 364 samples varying per country implemented with the Rolling Forecasting Origin resampling technique. Root mean square error was applied to select the optimal model using the smallest value. The final number of predictors tried at each split (mtry) used for each country model is 9 with 500 trees (for details on the code see GitHub at https://github.com/covidrealtime/covidrealtime).

The percentages of variance explained, i.e. the measure of how well out-of-bag predictions explain the target variance of the training set, varies between 82.68 and 95.47 for each country model, except for France and Finland with 65.04 and 65.26 percent of variance explained respectively.

We use the results of the RF models for Partial Dependence Plots (PDP) and for the Bump Chart (to compare feature importance ranks by countries) [55] in the Covid-Predictor-Tracker app. For validation purposes, the sensitivity analysis to finalize the parameters for our RF model covers several versions of the extent of time lag between predictors and reported infections and tests on dimensionality reduction, i.e., we produce new versions of restrictions by merging restrictions with partially relaxed measures (for example merging complete and partial closure of hotels and accommodation services). Further, we test different parameters of resampling time slices during model training.

### Hierarchical Clustering

To find the typical groups of countries with similar country characteristics, we perform a hierarchical cluster analysis, which is a common method to form country groups (for example [56, 57]). The variables included in the cluster analysis are time-constant, therefore this analysis is conducted only once.

Because we had no prior hypothesis on the number of clusters, and few covariates, we perform agglomerative hierarchical clustering with the stats [58] R package. The following variables are included in the clustering algorithm:

- population size,
- healthcare expenditures (in 1000 Euros per capita),
- cultural participation of 16 year-olds and older (percentage not attending any cultural event in the last 12 months),
- percentage of population in age groups (under 20, 20–39, 40–59, 60–79, above 80 years-old),
- percentage of males.

Again, variables are standardized before going into the model and in some instances scales are increased to give them a bigger weight in the cluster analysis. As an internal validation step, we multiply the scale of the variables “population size” and “percentage of males” with 1.1, and the scale of variables “healthcare expenditures” and “cultural participation” with 1.6. The aspects of choosing the exact magnitude of the weights are the maximization of the cophenetic correlation and the achievement of a sufficient number of clusters when defining the optimal number of clusters.

We use Euclidean distance measures to capture the dissimilarity between two countries. The distance between two clusters is measured with the Ward’s method [26], because in our analysis this method results in the highest (0.67) correlation between the cophenetic distances (height at which two clusters are combined) and the dissimilarity measures.

We define the optimal number of clusters with the average silhouette method. The silhouette width measures how close the points of a cluster are to the points of the neighboring cluster [26]. A high value of average silhouette width indicates that the observations are clustered well. A low value indicates the opposite, observations lying between or in the wrong clusters. The value with the highest average silhouette width is the optimal one for the clusters, 7 in our case.

As a result of hierarchical clustering the countries involved in the analysis are assigned to seven distinct clusters. The Nordic countries are allocated in the first cluster with the Netherlands, Belgium, and Austria. The Balkan countries and Hungary are in the second cluster. The third cluster comprises the Czech Republic and Slovenia, the fourth cluster Germany and France. The Mediterranean countries are assigned to the fifth cluster, and Poland and Slovakia form the sixth cluster. Ireland forms a separate cluster alone. We will provide more detail and a visualization in the result section.

### The Covid-Predictor-Tracker Interactive Dashboard

**FIGURE 2:**
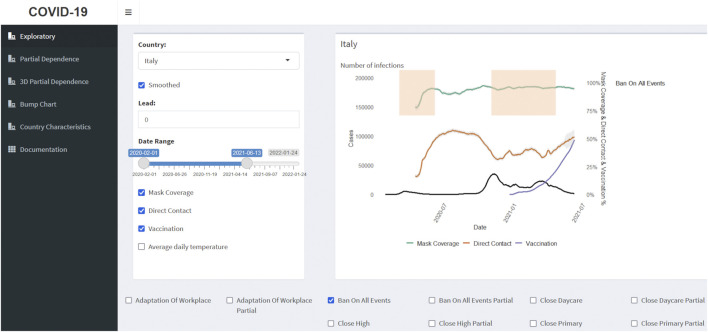
Covid-Predictor-Tracker Interface. Here, the interactive exploration page displays COVID-19 infections for Italy from 1 February 2020 till 13 June 2021, as well as reported mask coverage, direct contact, and vaccination rates. The red bars represent periods with interventions in place to limit all indoor/outdoor mass and public gatherings (Covid-Predictor-Tracker, Italy, 2020–2021).

Our interactive data visualization and analysis tool (see Figure 2) is created with the shiny [59] and shinydashboard [60] R packages. The inputs of the application are the prepared databases and the results of the RF models, described in the previous sections. We encourage readers to use the app https://corona.stat.uni-muenchen.de/covid_FI/ while reviewing the Results chapter.

## Results

Our results are based on data from 2/2020 to 1/2022 from nine different data sources, the COVID-19 cases from JHU CCSE, CTIS behavioral responses, weather info from NOAA, vaccination data from Our World in Data, response measures and variant info from ECDC, and Eurostat data on country characteristics on population, health expenditures, and cultural participation.

### COVID-19 Infection Predictors

**FIGURE 3:**
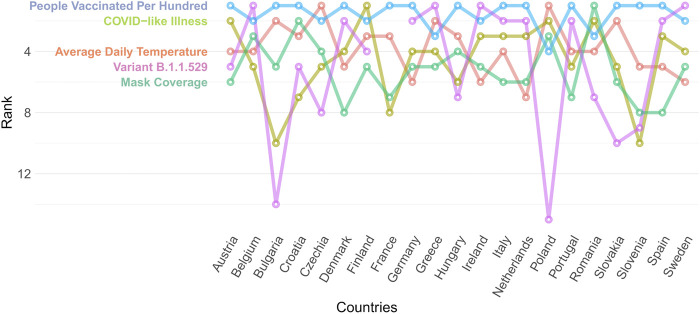
Rank position of top 5 predictors across selected countries of the European Union, random forest time series model from Covid-Predictor-Tracker (Covid-Predictor-Tracker, Selected countries of the European Union, 2020–2022).

Based on our RF time series model, the five strongest predictors overall of the daily new COVID-19 cases (see Figure 3) across selected countries in the EU (in descending order) are the percentage of people vaccinated at least once, the percentage of the B.1.1.529 variant (Omicron) by week, the average daily temperature of the given day, the share of people who self-assessed having COVID-like symptoms within the last 24 h, and the percentage of respondents self-reported using a mask. The importance rank positions of most predictors vary between countries. While the proportion of people vaccinated at least once is among the top 5 predictors in all analyzed countries, the predicting power of the other top predictors are more varied.

We learned from different combinations of PDPs that although the percentage of people vaccinated at least once is a powerful predictor in all countries, its effect on the change on daily new cases is ambiguous. A higher percentage of vaccinated people often goes with a moderate increase in the daily new COVID cases. This association might occur due to the emergence of new variants with different spread patterns since the start of the vaccination period. At the same time the other strong, but more dynamic predictor associated with the vaccination, the percentage of newly vaccinated people shows a negative effect of the vaccinations on the daily new COVID-19 cases.

**FIGURE 4:**
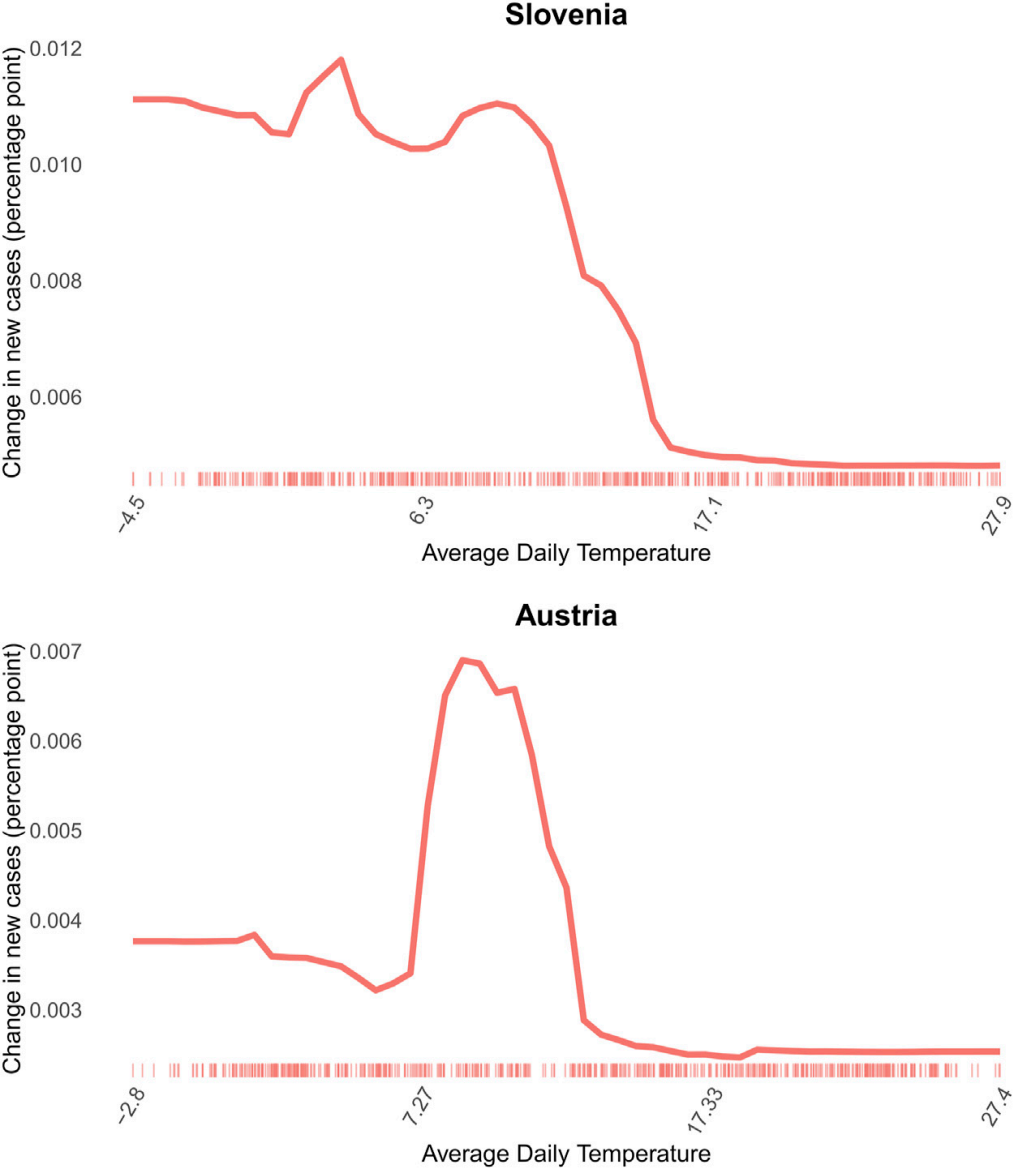
2D partial dependence of average daily temperature on daily new COVID-19 cases in Slovenia, example for rather steady association and Austria, example for staggered association (Covid-Predictor-Tracker, Slovenia, Austria, 2020–2022).

In many countries, such as in Italy and Slovenia, there is a steady slowdown in the increase of daily new cases as temperatures rise. In some other countries, for example in Austria, Hungary and Germany, the association is more staggered: the daily new cases start increasing strongly before the average daily temperature reaches 10°C, see Figure 4. As the average daily temperature reaches over 10°C, the increase of the percentage of population recorded with new COVID-19 infection is getting smaller in every country, showing a similar pattern as flu spread [61].

The share of the Omicron variant (B1.1.529) is one of the top 5 predictors in every country (between 2/2020 and 1/2022), except for some Eastern -and Central-European countries. This can be explained by the short time between the appearance of this variant and the end of our analysis. The effect of new vaccinations is higher in countries with low vaccination rates. These are countries with lower healthcare expenditures and lower population size, namely the Balkan countries, Czech Republic, and Slovakia. In these countries the percentage of vaccinated people is 51.27%, while in the other countries it is 76.52%.

**FIGURE 5:**
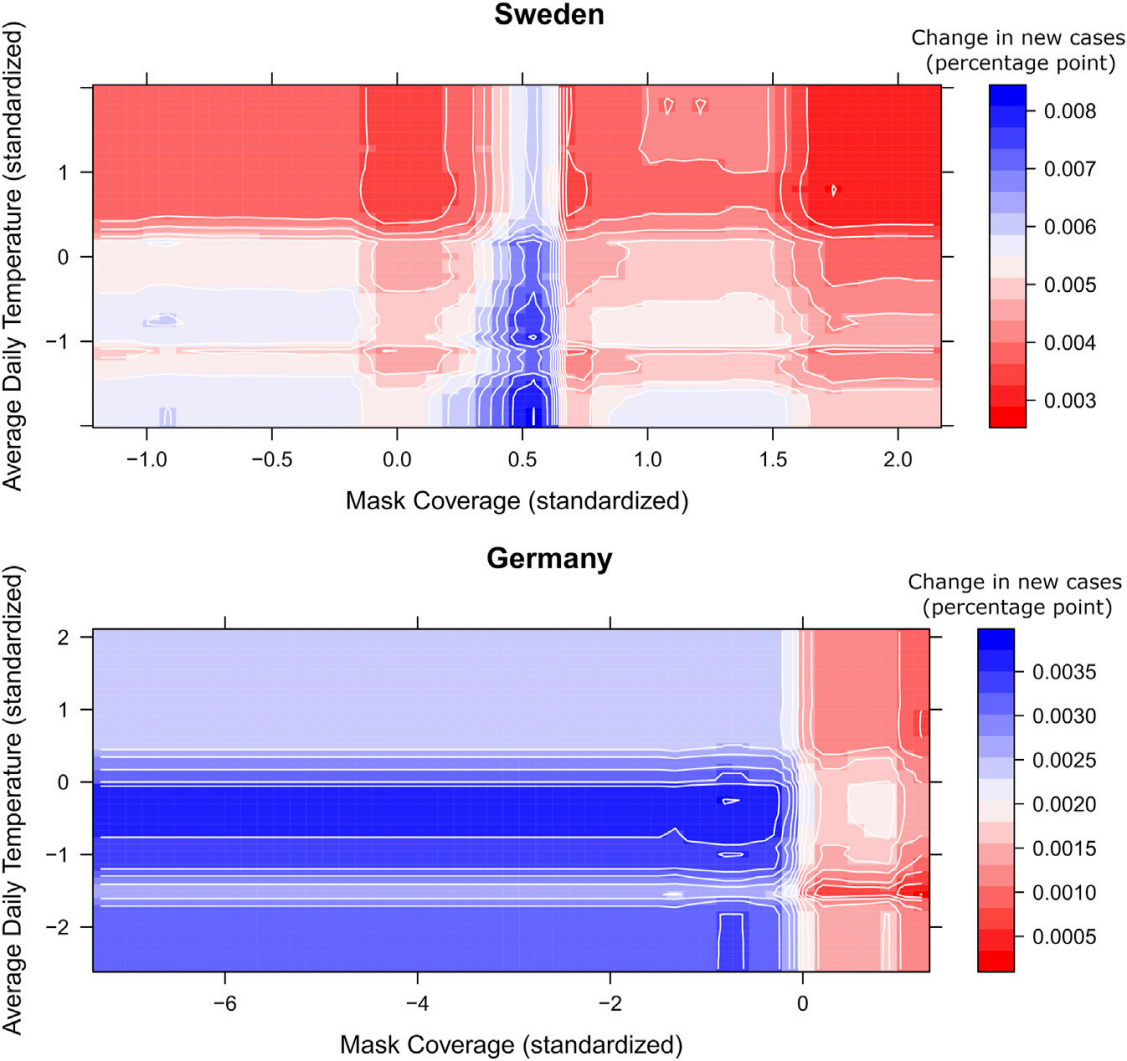
3D partial dependence of mask coverage and average daily temperature on daily new COVID-19 cases in Sweden, 3D partial dependence of mask coverage and new people vaccinated per hundred on daily new COVID-19 cases in Germany from Covid-Predictor-Tracker. The colors show the change in new cases in percentage points for each combination of mask coverage and average daily temperature (Covid-Predictor-Tracker, Sweden, Germany, 2020–2022).

The usage of protective masks is often among the top predictors (in countries where usage varied), having a negative effect on the daily new cases in most of the countries, both with colder and warmer average daily temperatures (see Figure 5). A higher percentage of reported usage of protective masks combined with more new vaccinations also contributed to the deceleration of the spread of COVID-19 in most countries, with some exceptions such as France, Greece, and Ireland.

Effects of restriction measures on the daily new COVID cases are more difficult to interpret because of their dependence on the pandemic levels. Nevertheless, we identified restrictive measures that had a negative effect on the daily new cases. We found the closure of non-essential shops, pubs, daycares, and primary schools to be associated with the decline of the spread of the pandemic in most countries. The importance of the closure of daycares and primary schools was the highest in the Balkan countries.

### Effects of Country Specific Characteristics on the COVID-19 Infection Predictor Importance Ranks

**FIGURE 6:**
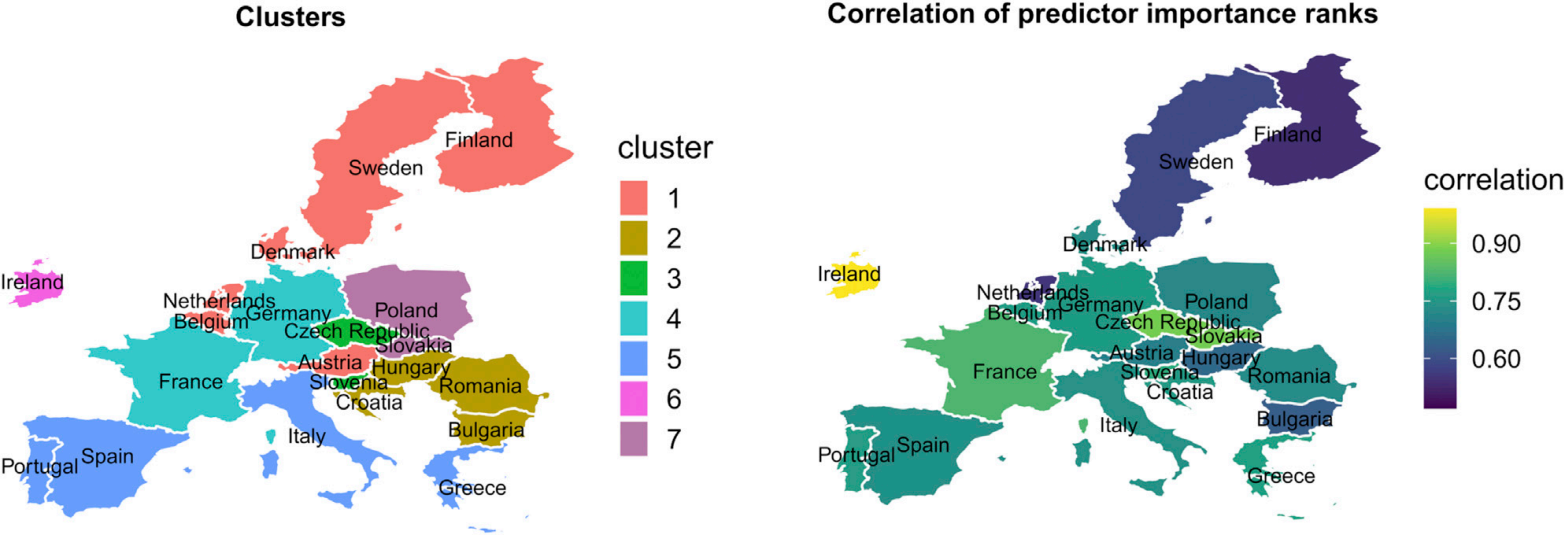
Country clusters and strength of the correlation of Random Forest predictor importance ranks between clusters and countries within the clusters from Covid-Predictor-Tracker (Covid-Predictor-Tracker, Selected countries of the European Union, 2020–2022).

Figure 6 displays the effect of time-constant country characteristics (like age, gender distribution, health expenditure, and cultural participation) on the RF predictor importance ranks on changes in the COVID-19 confirmed daily new infections. The correlation of COVID-19 predictor importance ranks between relevant country clusters and the single countries within the clusters are high. The correlations vary between 0.42 and 0.75.

The correlation is the weakest among the Nordic countries, followed by the Balkan countries. In all other clusters the correlation is middle/high. This means that the country characteristics, which formed these country clusters, might well determine the importance rank of a predictor in the countries of these clusters in general, i.e., the order of the variables that explain the virus spread.

## Discussion

Our main goal was to build one time series model and analyze the relative effects of various country characteristics, NPIs and other measures in the spread the COVID-19 infection. Most of the COVID-19 studies investigate the effect of different lock-down types [62, 63], vaccination and personal protective equipment separately, while we incorporated them into one model among many other predictors.

In line with our results, an observational study [64] shows the superior effect of vaccination over lock-downs in Israel. Conforming to our findings, high-rate mask usage is more beneficial than lock-downs alone [65] and mask usage precedes lock-down effects in a meta-analysis [66], though Sharma [67] shows that in more specific conditions some restrictions have greater effect than mask usage.

### Strengths

The Covid-Predictor-Tracker online interactive visualization tool belongs to the rare group of model-based analytical dashboards, as Ivanković et al. [12] state, which incorporates socioeconomic factors complementing COVID-19 predictors. Though COVID-19 RF prediction is not novel (see for example [13–15]), using this machine learning approach in a dashboard is unique. Alali et al. [23] show the superior performance of the inclusion of lagged data in machine learning models when the method is applied to time series data. We went further and apart from the inclusion of lagged data, we applied time series cross-validation to consider information from past data in order to improve our RF model.

The models and the interactive tool can help substantive researchers to reveal a more detailed image of the effect of country-level restriction measures. The open source code for the Covid-Predictor-Tracker allows continuous updates to the presentation and model, and with that allows continuous monitoring of the evolution of the pandemic and the effects of preventive measures. The effect of time-constant country characteristics can also be examined.

### Limitations

While the tool would allow us to do so, in this article we did not go into specific country-level analyses, nor precise focus of the included predictors is available compared to other analyses focusing on a specific theme and geography like Fukumoto et al. [68], who investigated the effect of school closures in Japan on the spread of COVID-19.

To improve our predictions, some transformations of the outcome variable (e.g., compound growth rate or growth curve slope estimates [69]) could be studied. We ran our model separately for each country and cluster but countries or clusters could be considered using spatial models as well. Statistical models such as Spatial Error Model, Spatial Lag Model [70] or Geographically Weighted Regression, or its extension into the machine learning approach, namely Geographically Weighted Random Forest [71] could be applied. The latter one is a local nonlinear nonparametric regression model considering topography, which integrates a spatial weight matrix into RF. Competing machine learning applications [72] for our research question might be Recurrent Neural Network and Long Short Term Memory or Gradient Boosted Machine [19]. The data about country-specific response measures have several limitations. There are differences in the implementation of these measurements between countries, for example in the enforcement of the restriction measures or exceptions to them. Regional measures within a country are not present in this dataset and delays in the implementation of the response measures are also possible [5].

### Conclusion

We found that the most important predictors of the daily new COVID-19 cases in the EU include proportion of vaccinated people, the spread of different variants, the average daily temperature, self-reported COVID-like symptoms, and the use of protective masks from 2/2020 to 1/2022. The effect of environmental and behavioral factors, vaccinations, emergence of new variants, and application of restrictive measures aiming to decelerate the spread of COVID-19 do have different effects in different countries. These predictors tend to have a more similar effect in countries with similar characteristics with respect to population size, cultural participation, healthcare expenditures, and population distribution by sex and age group. The emergence of the Omicron variant resulted in the highest increase in the Nordic countries and the Mediterranean. Moreover, new vaccinations are the most beneficial in countries with lower healthcare expenditures, and the effect of closing daycares and primary schools on reducing the increment of daily new cases is highest in the Balkan countries.
