# Supplementary material for: Real-Time Analysis of Predictors of COVID-19 Infection Spread in Countries in the European Union Through a New Tool
Source: Int J Public Health. 2022 Oct 6;67:1604974. doi: 10.3389/ijph.2022.1604974 (PMC9582119; doi:10.3389/ijph.2022.1604974)
Supplement: Supplementary file 1 [file DataSheet1.docx]

**Supplementary material**

*Real-time analysis of predictors of COVID-19 infection spread in*

*countries in the European Union through a new tool*

**Aniko Balogh, Anna Harman and Frauke Kreuter**

International Journal of Public Health

Table of contents

[Data sources and database description 1](#_Toc115124520)

[Time-constant country characteristics 1](#_Toc115124521)

[Time-varying COVID-19 related variables from various sources 3](#_Toc115124522)

[Code structure 9](#_Toc115124523)

[References to the R packages used for the Covid-Predictor-Tracker 18](#_Toc115124524)

# Data sources and database description

## Time-constant country characteristics

Name of database: **country_char**

| **1.Population** | Population of 1. January 2019 by age group and sex |
| --- | --- |
| **Data source** | ‘demo_pjangroup’ via eurostat R package (<https://cran.r-project.org/web/packages/eurostat/index.html>). The raw data is pulled from <https://ec.europa.eu/eurostat> |
| **Accessed** | 26 Jan. 2002 |
| geo | Geopolitical entity |
| Y_LT5 | Less than 5 years |
| Y5-9 | From 5 to 9 years |
| Y10-14 | From 10 to 14 years |
| Y15-19 | From 15 to 19 years |
| Y20-24 | From 20 to 24 years |
| Y25-29 | From 25 to 29 years |
| Y30-34 | From 30 to 34 years |
| Y35-39 | From 35 to 39 years |
| Y40-44 | From 40 to 44 years |
| Y45-49 | From 45 to 49 years |
| Y50-54 | From 50 to 54 years |
| Y55-59 | From 55 to 59 years |
| Y60-64 | From 60 to 64 years |
| Y65-69 | From 65 to 69 years |
| Y70-74 | From 70 to 74 years |
| Y75-79 | From 75 to 79 years |
| Y_GE75 | 75 years or over |
| Y80-84 | From 80 to 84 years |
| Y_GE80 | 80 years or over |
| Y_GE85 | 85 years or over |
| T | Total |
| M | Males |
| F | Females |

| **2.Health expenditures** | Total health care expenditure amount in millions of euro, 2018 |
| --- | --- |
| **Data source** | ‘hlth_sha11_hc’ via Eurostat R package, <https://cran.r-project.org/web/packages/eurostat/index.html> |
| **Accessed** | 26 Jan. 2002 |
| health_expenditures | Total health expenditures |

| **3.Cultural**  **participation** | Frequency of participation in cultural activities in the last 12 months by age, 2015. Percentage of those who did not attend any cultural event (cinema, live performances or cultural sites) in the last 12 months by age groups |
| --- | --- |
| **Data source** | ‘ilc_scp03’ via Eurostat R package, <https://cran.r-project.org/web/packages/eurostat/index.html> |
| **Accessed** | 26 Jan. 2002 |
| cult_Y_GE16 | 16 years or over |
| cult_Y_GE75 | 75 years or over |
| cult_Y16-24 | From 16 to 24 years |
| cult_Y16-29 | From 16 to 29 years |
| cult_Y25-34 | From 25 to 34 years |
| cult_Y25-64 | From 25 to 64 years |
| cult_Y35-49 | From 35 to 49 years |
| cult_Y50-64 | From 50 to 64 years |
| cult_Y65-74 | From 65 to 74 years |

## Time-varying COVID-19 related variables from various sources

Name of database: **tdata**

| **1.Testing** | Testing volume and positivity rate by week |
| --- | --- |
| **Data source** | European Centre for Disease Prevention and Control, Data on testing for COVID-19 by week and country,<https://www.ecdc.europa.eu/en/publications-data/covid-19-testing> |
| **Accessed** | 26 Jan. 2002 |
| country | Country name |
| country_code | 2-digit ISO country code |
| testing_new_cases | Number of new confirmed cases |
| tests_done | Number of tests done |
| testing_population |  |
| testing_rate | Testing rate per 100 000 population |
| testing_positivity_rate | Weekly test positivity (%): 100 x Number of new confirmed cases/number of tests done per week |

| **2.Response measures** | Non-pharmaceutical interventions taken by countries in response to the pandemics |
| --- | --- |
| **Data Source** | European Centre for Disease Prevention and Control, Data on country response measures to COVID-19 by week and country, <https://www.ecdc.europa.eu/en/publications-data/download-data-response-measures-covid-19> |
| **Accessed** | 26 Jan. 2002 |
| Country |  |
| date |  |
| year |  |
| week |  |
| AdaptationOfWorkplace | Adaptation of workplaces(e.g. to reduce risk of transmission |
| AdaptationOfWorkplacePartial | Adaptation of workplaces (e.g. to reduce risk of transmission)-partially relaxed measure |
| BanOnAllEvents | Interventions are in place to limit all indoor/outdoor mass/public gatherings |
| BanOnAllEventsPartial | Interventions are in place to limit all indoor/outdoor mass/public gatherings-partially relaxed measure |
| ClosDaycare. | Closure of educational institutions: daycare or nursery |
| ClosDaycarePartial | Closure of educational institutions: daycare or nursery -partially relaxed measure |
| ClosHigh | Closure of educational institutions: higher education |
| ClosHighPartial | Closure of educational institutions: higher education -partially relaxed measure |
| ClosPrim | Closure of educational institutions: primary schools |
| ClosPrimPartial | Closure of educational institutions: primary schools -partially relaxed measure |
| ClosPubAny | Closure of public spaces of any kind (including restaurants, entertainment venues, non-essential shops, partial or full closure of public transport, gyms and sport centers, etc) |
| ClosPubAnyPartial | Closure of public spaces of any kind (including restaurants, entertainment venues, non-essential shops, partial or full closure of public transport, gyms and sport centers etc) -partially relaxed measure |
| ClosSec | Closure of educational institutions: secondary schools |
| ClosSecPartial | Closure of educational institutions: secondary schools -partially relaxed measure |
| ClosureOfPublicTransport | Closure of public transport |
| ClosureOfPublicTransportPartial | Closure of public transport-partially relaxed measure |
| EntertainmentVenues | Closure of entertainment venues |
| EntertainmentVenuesPartial | Closure of entertainment venues-partially relaxed measure |
| GymsSportsCentres | Closure of gyms/sports centres |
| GymsSportsCentresPartial | Closure of gyms/sports centres-partially relaxed measure |
| HotelsAccommodation | Closure of hotels/accommodation services |
| HotelsAccommodationPartial | Closure of hotels/accommodation services-partially relaxed measure |
| IndoorOver100 | Interventions are in place to limit indoor mass/public gatherings of over 100participants |
| IndoorOver1000 | Interventions are in place to limit indoor mass/public gatherings of over 1000participants |
| IndoorOver1000Partial | Interventions are in place to limit indoor mass/public gatherings of over 1000participants-partially relaxed measure |
| IndoorOver100Partial | Interventions are in place to limit indoor mass/public gatherings of over 100participants-partially relaxed measure |
| IndoorOver50 | Interventions are in place to limit indoor mass/public gatherings of over 50participants |
| IndoorOver500 | Interventions are in place to limit indoor mass/public gatherings of over 500participants |
| IndoorOver500Partial | Interventions are in place to limit indoor mass/public gatherings of over 500participants-partially relaxed measure |
| IndoorOver50Partial | Interventions are in place to limit indoor mass/public gatherings of over 50participants-partially relaxed measure |
| MasksMandatoryAllSpaces | Protective mask use in all public spaces on mandatory basis (enforced by law) |
| MasksMandatoryAllSpacesPartial | Protective mask use in all public spaces on mandatory basis (enforced by law)-partially relaxed measure |
| MasksMandatoryClosedSpaces | Protective mask use in closed public spaces/transport on mandatory basis (enforced by law) |
| MasksMandatoryClosed  SpacesPartial | Protective mask use in closed public spaces/transport on mandatory basis (enforced by law)-partially relaxed measure |
| MasksVoluntaryAllSpaces | Protective mask use in all public spaces on voluntary basis (general recommendation not enforced) |
| MasksVoluntaryAllSpacesPartial | Protective mask use in all public spaces on voluntary basis (general recommendation not enforced)-partially relaxed measure |
| MasksVoluntaryClosedSpaces | Protective mask use in closed public spaces/transport on voluntary basis (general recommendation not enforced) |
| MasksVoluntaryClosedSpaces  Partial | Protective mask use in closed public spaces/transport on voluntary basis (general recommendation not enforced)-partially relaxed measure |
| MassGatherAll | Interventions are in place to limit mass/public gatherings (any interventions on mass gatherings up to 1000 participants included) |
| MassGatherAllPartial | Interventions are in place to limit mass/public gatherings (any interventions on mass gatherings up to 1000 participants included)-partially relaxed measure |
| NonEssentialShops | Closures of non-essential shops |
| NonEssentialShopsPartial | Closures of non-essential shops -partially relaxed measure |
| OutdoorOver100 | Interventions are in place to limit outdoor mass/public gatherings of over 100participants |
| OutdoorOver1000 | Interventions are in place to limit outdoor mass/public gatherings of over 1000participants |
| OutdoorOver1000Partial | Interventions are in place to limit outdoor mass/public gatherings of over 1000participants-partially relaxed measure |
| OutdoorOver100Partial | Interventions are in place to limit outdoor mass/public gatherings of over 100participants-partially relaxed measure |
| OutdoorOver50 | Interventions are in place to limit outdoor mass/public gatherings of over 50participants |
| OutdoorOver500 | Interventions are in place to limit outdoor mass/public gatherings of over 500participants |
| OutdoorOver500Partial | Interventions are in place to limit outdoor mass/public gatherings of over 500participants-partially relaxed measure |
| OutdoorOver50Partial | Interventions are in place to limit outdoor mass/public gatherings of over 50participants-partially relaxed measure |
| PlaceOfWorship | Closure of places of worship |
| PlaceOfWorshipPartial | Closure of places of worship-partially relaxed measure |
| PrivateGatheringRestrictions | Restrictions on private gatherings |
| PrivateGatheringRestrictionsPartial | Restrictions on private gatherings-partially relaxed measure |
| RegionalStayHomeOrder | Regional stay-at-home orders for the general population at least in one region(these are enforced and also referred to as 'lockdown') |
| RegionalStayHomeOrderPartial | Regional stay-at-home orders for the general population at least in one region (these are enforced and also referred to as 'lockdown')-partially relaxed measure |
| RestaurantsCafes | Closure of restaurants and cafes/bars |
| RestaurantsCafesPartial | Closure of restaurants and cafes/bars-partially relaxed measure |
| SocialCircle | Social circle/bubble to limit social contacts e.g. to limited number of households |
| SocialCirclePartial | Social circle/bubble to limit social contacts e.g. to limited number of households-partially relaxed measure |
| StayHomeGen | Stay-at-home recommendations for the general population (which are voluntary or not enforced) |
| StayHomeGenPartial | Stay-at-home recommendations for the general population (which are voluntary or not enforced) -partially relaxed measure |
| StayHomeOrder | Stay-at-home orders for the general population (these are enforced and also referred to as 'lockdown') |
| StayHomeOrderPartial | Stay-at-home orders for the general population (these are enforced and also referred to as 'lockdown') -partially relaxed measure |
| StayHomeRiskG | Stay-at-home recommendations for risk groups or vulnerable populations (such as the elderly, people with underlying health conditions, physically disabled people, etc.) |
| StayHomeRiskGPartial | Stay-at-home recommendations for risk groups or vulnerable populations (such as the elderly, people with underlying health conditions, physically disabled people, etc.) -partially relaxed measure |
| Teleworking | Teleworking recommendation |
| TeleworkingPartial | Teleworking recommendation or workplace closures -partially relaxed measure |
| WorkplaceClosures | Closures of workplaces |
| WorkplaceClosuresPartial | Closures of workplaces-partially relaxed measure |

| **3. Variant** | Distribution of variants by week and country that were dominant for at least 4 weeks in any of the analyzed countries |
| --- | --- |
| **Data source** | European Centre for Disease Prevention and Control, Data on SARS-CoV - variants in the EU/EEA,<https://www.ecdc.europa.eu/en/publications-data/data-virus-variants-covid-19-eueea> |
| **Accessed** | 26 Jan. 2002 |
| country |  |
| year |  |
| week |  |
| percent_variant.B.1.1.529 | Percent of variant B.1.1.529 |
| percent_variant.B.1.1.7 | Percent of variant B.1.1.7 |
| percent_variant.B.1.617.2 | Percent of variant B.1.617.2 |
| percent_variant.Other | Percent of other variants |

| **4. Weather** | Daily average temperatures are taken at weather stations closest to national capitals of the countries involved in the analysis |
| --- | --- |
| **Data source** | National Centers for Environmental Information,<https://www.ncdc.noaa.gov/> |
| **Accessed** | 26 Jan. 2002 |
| country_code | 2-digit ISO country code |
| date |  |
| tavg | average daily temperature, celsius |

| **5. Vaccianation** |  |
| --- | --- |
| **Data source** | Our World in Data,<https://ourworldindata.org/coronavirus> |
| **Accessed** | 26 Jan. 2002 |
| iso_code: ISO country code | ISO country code |
| country |  |
| date |  |
| total_vaccinations | total number of doses administered |
| people_vaccinated | total number of people who received at least one vaccine dose. If a person receives the first dose of a 2-dose vaccine, this metric goes up by 1. If they receive the second dose, the metric stays the same |
| people_fully_vaccinated | total number of people who received all doses prescribed by the vaccination protocol. If a person receives the first dose of a 2-dose vaccine, this metric stays the same. If they receive the second dose, the metric goes up by 1 |
| new_vaccinations | daily change in the total number of doses administered |
| new_vaccinations_smoothed | new doses administered per day (7-day smoothed (for countries that don't report data on a daily basis, the daily changes on doses assumed to be equal over the period in which no data was reported) |
| total_vaccinations_per_hundred | people vaccinated per 100 people in the total population of the country |
| people_vaccinated_per_hundred | people vaccinated per 100 people in the total population of the country |
| people_fully_vaccinated_per_  hundredpeople | fully vaccinated per 100 people in the total population of the country |
| new_vaccinations_smoothed_per_  million | daily vaccinations per 1,000,000 people in the total population of the country |

| **6. Covid cases** | Number of daily new COVID-19 cases, recoveries, deaths. |
| --- | --- |
| **Data source** | The raw data is pulled from the Johns Hopkins University Center for Systems Science and Engineering Coronavirus repository, <https://systems.jhu.edu/research/public-health/ncov/> via the coronavirus R package (<https://github.com/RamiKrispin/coronavirus>) |
| **Accessed** | 26 Jan. 2002 |
| cases_new | Confirmed daily new cases |
| deaths_new | Daily number of deaths |
| recovered_new | Daily number of the recovered |

| **7. CTIS** | Global COVID-19 Trends and Impact Survey |
| --- | --- |
| **Data source** | <https://covidmap.umd.edu/> |
| **Accessed** | 26 Jan. 2002 |
| fb_data.iso_code | ISO country codes |
| fb_data.percent_cli | weighted percentage of respondents that have reported Covid Like Illness |
| fb_data.cli_se | standard error of percent_cli |
| fb_data.percent_cli_unw | unweighted percentage of respondents that have reported CLI |
| fb_data.cli_se_unw | standard error of percent_cli_unw |
| fb_data.sample_size_cli | sample size for calculating CLI |
| fb_data.smoothed_cli | smoothed percentage of respondents that have reported Covid Like Illness |
| fb_data.smoothed_cli_se | standard error of smoothed percent_cli |
| fb_data.sample_size_smoothed_cli | sample size for calculating smoothed CLI |
| fb_data.percent_mc | weighted percentage of respondents that have reported using a mask |
| fb_data.mc_se | standard error of percent_mc |
| fb_data.percent_mc_unw: | unweighted percentage of respondents that have reported use mask cover |
| fb_data.mc_se_unw | standard error of percent_mc_unw |
| fb_data.sample_size_mc | sample size for calculating mask coverage |
| fb_data.smoothed_mc | smoothed percentage of respondents that have reported use mask cover |
| fb_data.smoothed_mc_se | standard error of smoothed percent_mc |
| fb_data.sample_size_mc_smoothed | sample size for calculating smoothed mc |
| fb_data.percent_dc | weighted percentage of respondents that have reported had direct contact (longer than one minute) with people not staying with them in last 24 hours |
| fb_data.mc_se_dc | standard error of fb_data.percent_mc |
| fb_data.percent_dc_unw | unweighted percentage of respondents that have reported use have direct contact with people not staying with them |
| fb_data.dc_se_unw | standard error of percent_dc_unw |
| fb_data.sample_size_dc | sample size for calculating direct contact |
| fb_data.smoothed_dc | smoothed percentage of respondents that have reported direct contact |
| fb_data.smoothed_dc_se | standard error of smoothed percent_dc |
| fb_data.sample_size_dc_smoothed | sample size for calculating smoothed dc |

# Code structure

All codes can be found in our [github](https://github.com/covidrealtime/covidrealtime) repository.

*data_collection folder*

 Create_database.R

- Creates the databases for the first time. Downloads data from all sources, creates and saves two databases:

- country_car: country characteristics (merged eurostat databases)

- tdata: data from all other sources, with time variable

- Uses the following scripts:

- data_collection/Save_data.R

- data_collection/Collect_data.R

- data_collection/Merge_data.R

Save_data.R

- Contains two functions (save database to local or to online location) to save databases.

- If the database should be saved online, the data will be written to password protected Google Sheets spreadsheet. In this case, an authentication file is necessary to reach the appropriate Google Sheets account. This file has to be located under a directory called “.secrets” in the working directory. The authentication file is provivided on Canvas.

- In both cases, if “archive” is set to true, if the database already exists, the old database will be kept and renamed.

Collect_data.R

- Downloads data from all sources.

- Filter time variable for latest eurostat databases:

- Demographics: 2019

- Number of practicing physicians: 2019

- Health expenditures: 2018

- Cultural participation: 2015

Merge_data.R

- Formats the data and merges it into two databases:

- country_car: country characteristics (merged eurostat databases)

- tdata: data from all other sources, with time variable

- Country characteristics

- Demographic variables:

- total population

- population by sex

- population by age groups (under 30, above 75)

- Health expenditures

- Cultural participation:

- Percentage of population by age groups who didn't attend on any cultural event in the last 12 months (cinema, live performances or cultural sites)

- Age groups: 16 years and older, under 30, above 75

- Data with time variable:

- Formats databases to enable merging them

- Keeps all variables

- Most data are per day (data on testing per week)

- Uses the following scripts:

- functions/Data_preparation_functions.R

- functions/Data_cleansing_functions.R

Update_data.R

- Updates tdata with new records since the last download and saves it.

- Date of the last day of data availability is often different for data sources and for countries within data sources. The data will be updated for every variable from the first day when data is not available for all the countries.

- Non-missing values stay the same.

- Uses the following scripts:

- functions/Data_preparation_functions.R

- data_collection/Save_data.R

Revise_data.R

- Recollects and merges tdata from all data sources. Saves the updated dataset.

- Lists the differences between the old and the new tdata.

- Functions are available to examine the differences:

- number of differences per variable

- first n differences per variable

- last n differences per variable

- all differences for one variable

- new variables that do not exist in the old tdata

- Functions are available to update the old tdata with the new values:

- update selected record of a variable

- update all different records of a variable

- add new variable to old tdata

- replace old tdata with new tdata

- Uses the following scripts:

- data_collection/Save_data.R

- data_collection/Collect_data.R

- data_collection/Merge_data.R

- data_collection/Data_revision_functions.R

*functions folder*

Data_preparation_functions.R

- Contains functions used during the preparation and merge of the data.

Data_cleansing_functions.R

- Contains functions for data cleansing.

Data_revision_functions.R

- Contains functions used during the data revision.

- Shows differences in details between two databases.

- Updates database.

Get_data.R

- Contains a function to load the databases form a local or online location.

- If the database should be loaded from Google Sheets, an authentication file is necessary to reach the appropriate Google Sheets account. This file has to be located under a directory called “.secrets” in the working directory. The authentication file is provivided on Canvas. The authentication file may be produced automatically, in this case in sake of reproductibility you have to overwrite the authentication file provided here with the file provided on Canvas (starting with dbea...).

RF_cluster_functions.R

- Contains functions to prepare data for RF modeling at cluster level

- Standardizes predictors

- Calculates time intervals for countries to be divisible with the length of the period horizon+window and trims data if necessary

- RF model for all clusters

- Calculates repeated feature importance for clusters

RF_functions.R

- Contains functions to prepare data for RF modeling for countries

- Standardizes predictors

- RF model for all the countries

- RF estimates for the Partial Dependence Plots

- Calculates repeated feature importance for countries

*helpers folder*

Near_stations.R

- Lists weather station IDs near to capitals.

- Normally the nearest station is selected.

- In some cases the nearest station is not functioning in the whole time interval, in these cases the station is selected manually from the next nearest stations.

Prepare_run.R

- Prepares the data collection and analysis.

- **Must be run before any other codes.**

- Sets the working directory and maximum date for data collection

- Creates a dataframe with the EU countries, capitals, different country codes, latitudes, longitudes and weather station IDs.

Data_cleansing.R

- Explores the data to discover the necessary data cleansing steps.

Add_variable_labels.R

- Adds labels to the variables.

Change_variable_types.R

- Change variable types (to numerical, factor or date).

Set_up_authentication.R

- Generates the token to access the private Google Sheets where the data are stored.

- Sets the directory where the generated token will be stored.

- Opens a browser and starts an interactive authentication to generate the token.

- Don’t run this without the authentication key. The purpose of this script here is only to demonstrate how the authentication file was created.

Get_and_prepare_data.R

- Loads the two (tdata and country_char) databases from Google Sheets.

- Adds variable labels and changes the variable types if necessary.

- Uses the following scripts:

- functions/Get_data.R

- helpers/Change_variable_types.R

- helpers/Add_variable_labels.R

Restriction_labels.R

- Contains the restriction labels and their descriptions to be displayed in the dashboard instead of their abbreviation.

*random_forest folder*

Cluster_RF.R

- Runs Random Forest models for each cluster:

- Creates a time-varying dbase adding cluster membership

- Preprocessing:

- selects variables for the model

- computes smoothing, leads, lags, etc

- standardises predictors, checks for highly correlated predictors

- calculates time intervals for countries to be divisible with the length of the period horizon+window

- Runs RF with fixed window timeslices on cumulative smoothed outcome on all countries separately

- Calculates repeated feature importance for each cluster

- Saves results as input for the rank correlation of the 'Country characteristics' tab

- Uses the following scripts:

- functions/RF_cluster_functions.R

Random_forest.R

- Runs Random Forest models for each country:

- Prepares the data and trains RF models on countries.

- Preprocessing:

- selects variables for the model

- computes smoothing, leads, lags, etc

- standardises predictors, checks for highly correlated predictors

- Runs RF with fixed window timeslices on cumulative smoothed outcome on all countries separately

- Gives the RF estimates for the Partial Dependence Plots

- Calculates repeated feature importance for countries

- Saves results and further input for the Shiny visualization

- Uses the following scripts:

- functions/RF_functions.R

rank_corr.R

- Creates rank variables per clusters

- Creates rank variables per countries

- Computes correlation between the repeated variable importance rank of the clusters and the repeated variable importance rank of the countries within clusters

- Saves results for the visualization 'Country characteristics tab'

*cluster folder*

Hierarchical_cluster.R

- Assigning countries to clusters with hierarchical clustering algorithms.

- Selects the variables for clustering,

- scales the variables,

- defines the optimal number of clusters,

- carries out the clustering and visualizes the results.

Cluster_Opt_Weight.R

- Finds the optimal combination of weights for distance measure by hierarchical clustering

- Optimal combination results in at least six clusters and highest cophenetic correlation

*shinydashboard folder*

app.R

- Contains the content and functionality of the shiny dashboard application.

- Defines the header, sidebar and tabs of the application.

- Uses the following scripts:

- shinydashboard/Shiny_prep_and_functions.R

- shinydashboard/Shiny_vis_functions.R

 Call_shiny.R

- Loads the data from googlesheets and prepares it.

- Prepares the data for the dashboard.

- Carries out RF and hierarchical clustering.

- Starts the application.

- Uses the following scripts:

- helpers/Get_and_prepare_data.R

- cluster/Hierarchical_cluster.R

- random_forest/Random_forest.R

- random_forest/Cluster_RF.R

- random_forest/rank_corr.R

- shinydashboard/Shiny_data_prep.R

Shiny_data_prep.R

- Prepares the data for the dashboard to enable fast visualization and to improve interactivity.

- Creates a list of databases for the countries for a more rapid selection.

- Creates a list of restriction measures applied in each country.

- Prepares the tooltips displayed in the dashboard.

- Creates input for the Partial Dependence Plots of the Random Forest train object for all countries (with FB data).

- Adds variables to help fasten the calculation of the coordinates of the restriction measures for the visualization.

- Uses the following script:

- helpers/Restriction_labels.R

Shiny_prep_and_functions.R

- Runs when starting the application

- Loads the data prepared in the previous steps when starting the shiny application.

- Contains functions to respond interactively to the queries in the shiny application, like

- calculating moving average,

- adding the selected lead to the dataset,

- calculating the coordinates for the visualization of the data and labels,

- adding tooltips to checkboxes,

- setting and resetting checkboxes.

Shiny_vis_functions.R

- Runs when starting the application

- Contains functions for the interactive visualization in the shiny application.

# References to the R packages used for the Covid-Predictor-Tracker

caret, Max Kuhn et al (2020). caret: Classification and Regression Training. R package version 6.0-86.[https://CRAN.R-project.org/package=caret](https://cran.r-project.org/package=caret)

compare, Paul Murrell (2015). compare: Comparing Objects for Differences. R package version 0.2-6. [https://CRAN.R-project.org/package=compare](https://cran.r-project.org/package=compare)

coronavirus, Rami Krispin and Jarrett Byrnes (2021). coronavirus: The 2019 Novel Coronavirus COVID-19 (2019-nCoV) Dataset. R package version 0.3.1. [https://CRAN.R-project.org/package=coronavirus](https://cran.r-project.org/package=coronavirus)

Cowplot, Claus O. Wilke (2020). cowplot: Streamlined Plot Theme and Plot Annotations for 'ggplot2'. R package version 1.1.1. [https://CRAN.R-project.org/package=cowplot](https://cran.r-project.org/package=cowplot)

data.table, Matt Dowle and Arun Srinivasan (2020). data.table: Extension of `data.frame`. R package version 1.13.6. [https://CRAN.R-project.org/package=data.table](https://cran.r-project.org/package=data.table)

dplyr, Hadley Wickham, Romain François, Lionel Henry and Kirill Müller (2020). dplyr: A Grammar of Data Manipulation. R package version 1.0.2. [https://CRAN.R-project.org/package=dplyr](https://cran.r-project.org/package=dplyr),

eurostat, (C) Leo Lahti, Janne Huovari, Markus Kainu, Przemyslaw Biecek. Retrieval and analysis of Eurostat open data with the eurostat package. R Journal 9(1):385-392, 2017. Version 3.6.84 Package URL:<http://ropengov.github.io/eurostat> Manuscript URL:<https://journal.r-project.org/archive/2017/RJ-2017-019/index.html>

factoextra, Alboukadel Kassambara and Fabian Mundt (2020). factoextra: Extract and Visualize the Results of Multivariate Data Analyses. R package version 1.0.7. [https://CRAN.R-project.org/package=factoextra](https://cran.r-project.org/package=factoextra)

googlesheets4, Jennifer Bryan (2020). googlesheets4: Access Google Sheets using the Sheets API V4. R package version 0.2.0. [https://CRAN.R-project.org/package=googlesheets4](https://cran.r-project.org/package=googlesheets4)

gridextra, Baptiste Auguie (2017). gridExtra: Miscellaneous Functions for "Grid" Graphics. R package version 2.3. [https://CRAN.R-project.org/package=gridExtra](https://cran.r-project.org/package=gridExtra)

Hmisc, Frank E Harrell Jr, with contributions from Charles Dupont and many others. (2020). Hmisc: Harrell Miscellaneous. R package version 4.4-2. [https://CRAN.R-project.org/package=Hmisc](https://cran.r-project.org/package=Hmisc)

httr, Hadley Wickham (2020). httr: Tools for Working with URLs and HTTP. R package version 1.4.2. [https://CRAN.R-project.org/package=httr](https://cran.r-project.org/package=httr)

iml, Molnar C, Bischl B, Casalicchio G (2018). “iml: An R package for Interpretable Machine Learning.” *JOSS*, **3**(26), 786.<https://joss.theoj.org/papers/10.21105/joss.00786>. R package version 0.10.1 [https://CRAN.R-project.org/package=iml](https://cran.r-project.org/package=iml)

jsonlite, Jeroen Ooms (2014). The jsonlite Package: A Practical and Consistent Mapping Between JSON Data and R Objects. arXiv:1403.2805 [stat.CO] URL<https://arxiv.org/abs/1403.2805>.

lubridate, Garrett Grolemund, Hadley Wickham (2011). Dates and Times Made Easy with lubridate. Journal of Statistical Software, 40(3), 1-25. URL<https://www.jstatsoft.org/v40/i03/>.

maps, Original S code by Richard A. Becker, Allan R. Wilks. R version by Ray Brownrigg. Enhancements by Thomas P Minka and Alex Deckmyn. (2018). maps: Draw Geographical Maps. R package version 3.3.0. [https://CRAN.R-project.org/package=maps](https://cran.r-project.org/package=maps)

pdp, Brandon M. Greenwell (2017). pdp: An R Package for Constructing Partial Dependence Plots. The R Journal, 9(1), 421--436. URL<https://journal.r-project.org/archive/2017/RJ-2017-016/index.html>

randomForest, Leo Breiman, Adele Cutler, Andy Liaw , Matthew Wiener (2018).

randomForest: Breiman and Cutler's Random Forests for Classification and Regression. R package version 4.6-14. [https://CRAN.R-project.org/package=randomForest](https://cran.r-project.org/package=randomForest)

ranger, Marvin N. Wright, Andreas Ziegler (2017). ranger: A Fast Implementation of Random Forests for High Dimensional Data in C++ and R. Journal of Statistical Software, 77(1), 1-17. doi:10.18637/jss.v077.i01

RColorBrewer, Erich Neuwirth (2014). RColorBrewer: ColorBrewer Palettes. R package version 1.1-2. [https://CRAN.R-project.org/package=RColorBrewer](https://cran.r-project.org/package=RColorBrewer)

readr, Hadley Wickham and Jim Hester (2020). readr: Read Rectangular Text Data. R package version 1.4.0. [https://CRAN.R-project.org/package=readr](https://cran.r-project.org/package=readr)

reshape2, Hadley Wickham (2007). Reshaping Data with the reshape Package. Journal of Statistical Software, 21(12), 1-20. URL<http://www.jstatsoft.org/v21/i12/>

rnoaa, Scott Chamberlain, Daniel Hocking, Brooke Anderson , Maëlle Salmon , Adam Erickson, Nicholas Potter, Joseph Stachelek, Alex Simmons, Karthik Ram, Hart Edmund, (2021). rnoaa: 'NOAA' Weather Data

from R. R package version 1.3.0. [https://CRAN.R-project.org/package=rnoaa](https://cran.r-project.org/package=rnoaa)

rvest, Hadley Wickham (2020). rvest: Easily Harvest (Scrape) Web Pages. R package version 0.3.6. [https://CRAN.R-project.org/package=rvest](https://cran.r-project.org/package=rvest)

shiny, Winston Chang, Joe Cheng, JJ Allaire, Carson Sievert, Barret Schloerke, Yihui Xie, Jeff Allen, Jonathan McPherson, Alan Dipert and Barbara Borges (2021). shiny: Web Application Framework for R. R package version 1.6.0. [https://CRAN.R-project.org/package=shiny](https://cran.r-project.org/package=shiny)

shinyBS, Eric Bailey (2015). shinyBS: Twitter Bootstrap Components for Shiny. R package version 0.61. [https://CRAN.R-project.org/package=shinyBS](https://cran.r-project.org/package=shinyBS)

shinydashboard, Winston Chang and Barbara Borges Ribeiro (2018). shinydashboard: Create Dashboards with 'Shiny'. R package version 0.7.1. [https://CRAN.R-project.org/package=shinydashboard](https://cran.r-project.org/package=shinydashboard)

sjlabelled, Lüdecke D (2020). _sjlabelled: Labelled Data Utility Functions (Version 1.1.7)_. doi: 10.5281/zenodo.1249215 (URL: https://doi.org/10.5281/zenodo.1249215), <URL: [https://CRAN.R-project.org/package=sjlabelled](https://cran.r-project.org/package=sjlabelled)

stats, R Core Team (2020). R: A language and environment for statistical computing. R Foundation for Statistical Computing, Vienna, Austria. R package version 4.0.0. [https://www.R-project.org/](https://www.r-project.org/)

stringr, Hadley Wickham (2019). stringr: Simple, Consistent Wrappers for Common String Operations. R package version 1.4.0. [https://CRAN.R-project.org/package=stringr](https://cran.r-project.org/package=stringr)

tidyverse, Wickham et al. (2019). Welcome to the tidyverse. Journal of Open Source Software, 4(43), 1686,

<https://doi.org/10.21105/joss.01686>

zoo, Achim Zeileis and Gabor Grothendieck (2005). zoo: S3 Infrastructure for Regular and Irregular Time Series. Journal of Statistical Software, 14(6), 1-27.

<https://doi.org/10.18637/jss.v014.i06>
